# A randomized, open, two-period, cross-over, single oral dose, pharmacokinetics and bioequivalence study of vitamin K_1_ in healthy volunteers under fasting and fed conditions

**DOI:** 10.3389/fphar.2026.1802709

**Published:** 2026-05-13

**Authors:** Yan Li, Yu Wang, Lu Qi, Yinjuan Li, Wenjing Zhong, Xinghe Wang

**Affiliations:** 1 Phase I Clinical Trial Center, Beijing Shijitan Hospital, Capital Medical University, Beijing, China; 2 Hainan Brilliant Pharmaceutical Co., Ltd., Chengdu, China

**Keywords:** bioequivalence, healthy volunteers, pharmacodynamics, pharmacokinetics, vitamin K1 injection

## Abstract

**Objective:**

Vitamin K_1_ (VK_1_) supplementation is an important therapeutic agent for the treatment of hemorrhage due to VK_1_ deficiency. It can be administered orally, intramuscularly, or via intravenous injection. This study was conducted to evaluate the bioequivalence of VK_1_ micelle produced by Hainan Biotech Pharmaceutical Co., Ltd. compared to the original drug KONAKION®MM when administered orally.

**Methods:**

This was a randomized, open-label, two-period, single-dose, crossover bioequivalence study conducted under both fasting and fed conditions, with 28 participants in each condition. Participants received 10 mg oral dose of test (T) or reference (R) drug in the first period and the opposite drug in the second period, with a 9-day washout. Blood concentrations of the E and Z isomers of VK_1_ were measured at 26 or 29 time points for fasting condition or fed condition, respectively. Bioequivalence was determined if the 90% confidence intervals (CIs) of the geometric mean ratios (GMRs) of the peak concentration (C_max_), the area under the concentration-time curve from time zero to the last measurable concentration (AUC_0–t_) and the extrapolated area under the curve from time zero to infinity ( ${\text{AUC}}_{0-\infty }$ ) after log transformation for VK_1_ E isomer fell within the 80.00%–125.00% range under both fasting and fed conditions. The International Normalized Ratio (INR) was used as an exploratory endpoint to observe changes following dosing. Safety assessments included vital signs and electrocardiograms (ECGs).

**Results:**

Under fasting conditions, the 90% CIs for the GMRs of C_max_, AUC_0-t_, and ${\text{AUC}}_{0-\infty }$ for the VK_1_ E isomer were 99.17%–119.24%, 103.91%–121.13%, and 105.72%–122.77%, respectively. Under fed conditions, the corresponding 90% CIs were 83.56%–120.82%, 84.98%–124.63%, and 83.97%–124.32%. All values fell within the 80.00%–125.00% equivalence margin, confirming bioequivalence between the two formulations. There were no significant changes in INR. The safety profile was favorable, with no serious adverse events (SAEs) or grade 3 or higher adverse events (AEs) reported.

**Conclusion:**

The VK_1_ micelle produced by Hainan Brilliant Pharmaceutical Co., Ltd. is bioequivalent to the original drug KONAKION®MM, supporting its approval as a generic product by oral administration. It demonstrated good safety and tolerability in healthy Chinese subjects.

**Clinical Trial Registration:**

http://www.chinadrugtrials.org.cn/index.html, identifier CTR20223408; https://www.chictr.org.cn/, identifier ChiCTR2500096211.

## Introduction

Vitamin K is a group of 2-methyl-1,4-naphthoquinone and its derivatives, mainly categorized into forms K_1_ and K_2_ in nature. In hepatocytes, within the endoplasmic reticulum, vitamin K is reduced to vitamin K hydroquinone, which participates in the carboxylation of specific glutamate residues on the precursor proteins of vitamin K-dependent coagulation factors, converting them into gamma-carboxyglutamic acid. This process enables these precursor proteins to form biologically active clotting factors II, VII, IX, and X. When vitamin K is deficient, the precursor proteins of these clotting factors cannot be carboxylated and remain as inactive proteins, leading to coagulopathy and bleeding. Vitamin K deficiency can result in multiple hemorrhagic disorders in newborns, impacting their health, and potentially causing disability or death. Thus, vitamin K deficiency poses a serious risk to neonates and infants, and vitamin K_1_ plays a crucial clinical role in preventing hemorrhagic diseases in newborns (Ipema, 2012; McPherson, 2020; Perrone et al., 2024; Natarajan et al., 2025). Warfarin is a dicoumarin anticoagulant, which is mainly used for anticoagulation and prevention of atrial fibrillation, venous thromboembolism and pulmonary embolism in clinic. But its most common and serious side effect is bleeding caused by drug overdose. In view of this adverse reaction, the main rescue drug is vitamin K_1_ (Lubetsky et al., 2003; Eichinger, 2016; Crowther et al., 2010; Zhang et al., 2021).

Vitamin K is lipophilic and can form mixed micelles with bile salts, thereby enabling absorption by enterocytes. Within the intestinal epithelial cells, vitamin K is further packaged into chylomicrons and then transported directly into the systemic circulation via the lymphatic system. The catabolism of vitamin K_1_ begins with CYP4F2-mediated initial ω-hydroxylation, followed by shortening of the polyisoprenyl side chain through β-oxidation to form carboxylic acids, which are conjugated with glucuronic acid and excreted in urine and bile (Mladěnka et al., 2022). The bioavailability of oral vitamin K_1_ (VK_1_) solution is higher than that of oral vitamin K_1_ tablets (van Rein et al., 2014). After oral administration of 2 mg of VK_1_ in healthy individuals, the t_max_ of VK_1_ is approximately 200 min, the C_max_ is about 29 ng/mL, and the t_1/2_ is around 360 min (Marinova et al., 2013).

In this study, the original vitamin K_1_ injection was developed by Roche with the brand name KONAKION®MM, with formulations of 0.2 mL: 2 mg and 1 mL: 10 mg. The 0.2 mL: 2 mg formulation is primarily used for neonatal and infant care, for the prevention and treatment of vitamin K deficiency bleeding (VKDB) in newborns and infants (Mihatsch et al., 2016). The 1 mL: 10 mg formulation is mainly used to treat bleeding caused by vitamin K deficiency and to prevent vitamin K deficiency in individuals who cannot obtain it nutritionally. KONAKION®MM has not been approved in China, and there are no locally produced original products or internationally recognized imported or locally produced equivalents available. Thus, we conducted a study to evaluate the bioequivalence (BE) between a generic vitamin K_1_ micelle developed by Hainan Brilliant Pharmaceutical Co., Ltd. and KONAKION®MM. Both formulations are micelles. Although the dosage form is an injection, the primary excipient is lecithin, which causes no significant irritation to the gastrointestinal mucosa and can be absorbed by the digestive tract. Therefore, both formulations can be administered either intravenously or orally. This study specifically evaluated the bioequivalence of oral administration under both fasting and fed conditions. Since VK_1_ is lipid-soluble, food is expected to influence drug absorption. Thus, this study was conducted under both fasting and fed conditions to meet the regular requirement of BE study guidance.

## Methods

### Ethics statement of human rights

The study was registered on the Chinese Clinical Trial website (http://www.chinadrugtrials.org.cn/index.html, number: CTR20223408, date: 23 December 2022). The bioequivalence clinical trial was retrospectively registered with the Chinese Clinical Trial Registry, which is recognized by the WHO (https://www.chictr.org.cn/, number: ChiCTR2500096211, date: 20 January 2025). The study was conducted at the Phase I Clinical Trial Center of Beijing Shijitan Hospital, Capital Medical University, from 29 December 2022, to 12 February 2023. The study protocol and informed consent form were approved by the Ethics Committee of Beijing Shijitan Hospital, Capital Medical University (Ethics Approval Number 2022(43)). All participants provided written informed consent after being fully informed about the study procedures prior to screening.

### Study design

This study was a randomized, open-label, two-period, single-dose, crossover bioequivalence trial conducted under both fasting and fed conditions. Twenty-eight participants for each fasting and fed studies were randomly assigned to Sequence A (TR) or Sequence B (RT) according to the order of their screening, with 14 participants in each sequence. T was for the test formulation (produced by Hainan Brilliant Pharmaceutical Co., Ltd.) and R was for the reference formulation (KONAKION®MM). The randomization code was generated using SAS version 9.4 by blocked randomization (block number: 4), with an allocation ratio of 1:1 between groups. A washout period of 9 days was set between the two periods to ensure that it exceeded seven elimination half-lives of the drug.

### Participants

Healthy subjects aged 18 years or above were eligible to participate in the screening process for this study. Male participants must weigh at least 50.0 kg, and female participants must weigh at least 45.0 kg, with a Body Mass Index (BMI) within the range of 19.0–26.0 kg/m^2^. Subjects were excluded if they met any of the following criteria: have a known allergy to vitamin K_1_ injection, its related compounds, or any of the excipients; have a history of severe illness; have undergone recent surgery; have a history of drug abuse; heavy smokers or alcoholics; be pregnant or suspected of being pregnant.

### Interventions

The test formulation (T) was Vitamin K_1_ Injection, with a specification of 1 mL:10 mg. Batch number: A2203101, potency: 98.3%, manufactured on 10 March 2022, and with an expiration date of February 2025. It was produced and provided by Hainan Brilliant Pharmaceutical Co., Ltd. The reference formulation (R) was Vitamin K_1_ Injection (brand name: KONAKION®MM), with a specification of 1 mL:10 mg. Batch number: F4080F02, potency: 95.9%, with an expiration date of March 2023. It was manufactured by Cheplapharm Arzneimittel GmbH. All doses administered to participants were from the same batch. The excipient composition for both formulations was soybean phosphatidylcholine, glycocholic acid, sodium hydroxide, hydrochloric acid and water for injection. The active ingredients and prescription types of the test formulation were consistent with those of the reference formulation. The Z-isomer content in both formulations was specified to be below 10%, which was consistent with the stability and quality control specifications. Both formulations were stored under identical conditions (protected from light, at controlled room temperature) and were handled in the same manner throughout the study.

For the study under fasting condition, participants received either the test formulation or the reference formulation in a fasting state during each period. The dose for each administration was 1 mL:10 mg, which was taken orally with 240 mL of water. Each participant received 10 mg single dose per period. For the study under fed condition, the randomization and dosing procedures for the 28 participants were identical to those under fasting conditions, but the only difference was that they took a standard high-fat breakfast 30 min before administration. The total caloric content of the high fat breakfast was approximately 900 kcal, of which fat accounted for about 50% of the total calories and carbohydrates accounted for approximately 30%. In parallel, the intake of green leafy vegetables was controlled for each meal so that the total daily vitamin K intake was approximately 100 μg, in order to minimize the impact of dietary VK_1_ on PK (Gijsbers et al., 1996; Jones et al., 2009). Subjects were hospitalized continuously for at least 48 h before dosing and for at least 48 h after dosing. During hospitalization, the use of concomitant medications, supplements (including vitamin K), alcohol, or tobacco was not permitted.

### Study endpoints

VK_1_ exists in both cis and trans configurations, with the trans-configuration (E-isomer) being responsible for its primary pharmacological activity, while the cis-configuration (Z-isomer) exhibits negligible pharmacological activity. In this study, the content of Z-isomer in both formulations was below 10%. Since the E isomer is the pharmacologically active component, the primary pharmacokinetic (PK) parameters were defined as the peak concentration (C_max_), the area under the concentration-time curve from time zero to the last measurable concentration (AUC_0–t_) and the extrapolated area under the curve from time zero to infinity ( ${\text{AUC}}_{0-\infty }$ ) for the changes from baseline of Vitamin K_1_ E isomer. Secondary PK parameters included the time to reach peak concentration (T_max_), the terminal half-life (t_1/2z_), the apparent volume of distribution during the terminal phase (V_z_/F), the apparent clearance during the terminal phase (CL_z_/F), the terminal elimination rate constant (λ_z_), and the percentage of the ${\text{AUC}}_{0-\infty }$ that was extrapolated beyond the last quantifiable concentration (AUC__%Extrap_) for the changes from baseline of Vitamin K_1_ E isomer, as well as the C_max_, AUC_0-t_, ${\text{AUC}}_{0-\infty }$ , T_max_, t_1/2 z_, V_z_/F, CL_z_/F, λ_z_, and AUC__%Extrap_ for the changes from baseline of Z isomers of Vitamin K_1_. Given that Vitamin K_1_ is an endogenous compound, the relevant guidelines for endogenous substances require the subtraction of background levels (FDA, 2015). The calculation formula for the change from baseline post-dose plasma concentration is: post-dose concentration minus the average of pre-dose concentrations at −48, −42, −36, −30, −24, −18, −12, −6, and 0 h, with any negative change values set to zero. Additionally, this study included an exploratory endpoint of INR to evaluate the trend of INR changes following oral administration of 10 mg of Vitamin K_1_. The maximum drug effect (E_max_), the effect-time curve from time zero to 48 h (AUEC_0–48h_), and the time to reach maximum effect (T_Emax_) for INR were calculated.

### PK and INR assessments

In the study of fasting condition, a total of 26 venous blood samples were collected in each period, with blood drawn at the following time points: 48, −42, −36, −30, −24, −18, −12, −6, and 0 h (within 1 h before dosing) and post-dose at 1, 2, 3, 4, 4.5, 5, 5.5, 6, 6.5, 7, 7.5, 8, 9, 10, 12, 24, and 48 h. At each time point, 3 mL of whole blood was collected into a blood collection tube containing heparin sodium. In the study of fed condition, 29 blood samples were collected at −48, −42, −36, −30, −24, −18, −12, −6, and 0 h (within 1 h before dosing) and post-dose at 1, 1.5, 2, 2.5, 3, 3.5, 4, 4.5, 5, 5.5, 6, 6.5, 7, 7.5, 8, 9, 10, 12, 24, and 48 h. Additionally, for INR measurement, 3 mL of whole blood was collected at 0 h (within 1 h before dosing) and post-dose at 1, 3, 6, 12, 24, and 48 h in each period for both studies under fasting and fed conditions. INR measurements were performed using a standardized coagulation analyzer. The normal reference range for INR in healthy adults was 0.8–1.2. Statistical analysis of INR endpoints, including AUEC_0–48h_, change from baseline, and T_Emax_, was performed using descriptive statistics. The pharmacodynamic endpoints were interpreted as supportive evidence of the absence of clinically meaningful effects on coagulation following a single oral dose of 10 mg VK_1_ in healthy subjects.

For PK samples, after centrifugation at 2 °C–8 °C, 1700 g for 10 min,the plasma was divided into testing and backup samples, which were then stored at −80 °C. The testing samples were sent to Chengdu Finelyse Pharmaceutical Technology Co., Ltd. (Chengdu, Sichuan, China) for drug concentration analysis. This study utilized HPLC-MS/MS to determine the concentrations of Vitamin K_1_, including both the E isomer (trans configuration) and Z isomer (cis configuration). Vitamin K_1_-d7 was used as the internal standard. Sample preparation involved protein precipitation with methanol, followed by liquid-liquid extraction. Calibration curves were fitted using a linear regression model with 1/x^2^ weighting. The linearity range for Vitamin K_1_ E isomer was 2.000–4,000 ng/mL, and for the Z isomer was 2.381–635.0 ng/mL. The interday precision coefficient of variation (CV%) for Vitamin K_1_ E isomer was <11.27%, and the accuracy ranged within 5.86%–12.38%. The interday precision coefficient of variation (CV%) for Vitamin K_1_ Z isomer was <7.34%, and the accuracy ranged within 4.90%–8.39%. Acceptance criteria for calibration standards required accuracy within ±15% (±20% at the lower limit of quantitation), and quality control (QC) samples were required to be within ±15% of nominal concentrations in at least two-thirds of QC samples. Matrix effects were evaluated using post-column infusion and were found to be minimal. Stability studies demonstrated that the analytes were stable under bench-top conditions (6 h), after three freeze-thaw cycles, and during long-term storage at −80 °C for 90 days. Incurred sample reanalysis (ISR) was performed on 10% of study samples, and the results met the acceptance criteria of two-thirds of repeats within ±20% of the initial value.

### Safety assessments

Any adverse events (AEs) occurring during the clinical study were observed in all subjects and assessed through vital signs measurements, physical examinations, laboratory tests, electrocardiogram evaluations, and adverse event reporting. Adverse events were classified according to CTCAE 5.0 and coded according to MedDRA 26.0. Adverse reactions (ADR) were defined as adverse events that were definitely related, probably related or possibly related to the study drug. All AEs were followed up until resolution or return to baseline levels.

### Statistical methods

Equivalence analysis was conducted using a linear mixed-effects model by SAS (version 9.4), with treatment, period, and sequence as fixed effects and subject within sequence as a random effect. Carryover effects were not included in the model, as no significant carryover was detected based on pre-dose concentration analysis. The log-transformed changes from baseline of C_max_, AUC_0-t_, and ${\text{AUC}}_{0-\infty }$ for the Vitamin K_1_ E isomer were analyzed and the 90% confidence intervals (CIs) for the GMRs (test formulation/reference formulation) of the primary endpoints were calculated. The statistical hypothesis for establishing bioequivalence followed the two one-sided tests (TOST) procedure. The null hypothesis (H_0_) for each primary PK parameter (C_max_, AUC_0–t_, ${\text{AUC}}_{0-\infty }$ ) was that the formulations were not bioequivalent (i.e., the true GMR is ≤ 80% or ≥125%). The alternative hypothesis (H_1_) was that the formulations are bioequivalent (i.e., the true GMR lies entirely within 80%–125%). Rejection of H_0_ at a significance level (α) of 0.05 for each one-sided test supports the conclusion of bioequivalence. The sample size for this study was determined using PASS software (version 14.0). Based on a pilot study, the within-subject coefficient of variation (CV) for the corrected E isomer’s C_max_, AUC_0-t_, and ${\text{AUC}}_{0-\infty }$ was 19.36%, 20.10%, and 20.16%, respectively, while for the corrected Z isomer, it was 19.94%, 18.71%, and 18.63%, indicating a within-subject variability of approximately 20%. Assuming a within-subject CV of 20%, a geometric mean ratio (GMR) of 0.93 for the test and reference formulations based on the pilot study, and a significance level of α = 0.05 with β = 0.2 (power = 80%), and a bioequivalence interval of 80.00%–125.00%, the calculated sample size was 23 subjects for a two-period, crossover design. Considering a 20% dropout rate, 28 healthy subjects were enrolled for both the fasting and fed studies. The Z isomer served as supportive evidence. The datasets were divided into the Full Analysis Set (FAS), Safety Set (SS), Pharmacokinetic Concentration Set (PKCS), Pharmacokinetic Parameter Set (PKPS), Bioequivalence Set (BES), and Pharmacodynamic Set (PDS). Subjects who experienced vomiting within twice the median T_max_ after receiving the investigational product were excluded from the PKPS and BES for that period. For subjects who withdrew before the second period, only data from the first period were included in the PKPS and BES.

## Results

### Subject disposition and baseline characteristics

**FIGURE 1:**
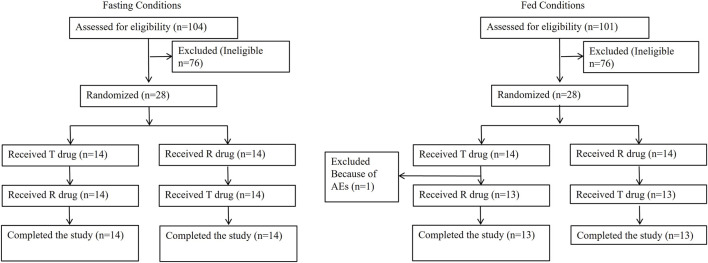
The subjects disposition flow diagram.

In the fasting study, 104 subjects were screened, and 28 subjects were enrolled, with 14 subjects in each of the TR and RT sequences, all of whom completed the study. For the fed study, 101 subjects were screened, and 28 subjects were enrolled. One subject withdrew due to an adverse event (AE) of acute gastroenteritis during the washout period after the first period, resulting in 27 subjects completing both periods. The subject who withdrew was from the TR sequence and did not receive the second-period dose. All other subjects received dosing for both periods, and there were no abnormalities during the high-fat meal consumption or the dosing process. One subject in the second period (RT sequence) experienced vomiting 19 min post-dose, which was within twice the median T_max_ time, while no other subjects experienced vomiting after dosing. The subjects disposition flow diagram is presented in Figure 1. The demographics and subject characteristics at baseline are presented in Table 1.

**TABLE 1:** The demographics and subject characteristics at baseline.

Parameters (Mean ± SD)	Fasting condition			Fed condition		
	Sequence A (TR) (N = 14)	Sequence B (RT) (N = 14)	Total (N = 28)	Sequence A (TR) (N = 14)	Sequence B (RT) (N = 14)	Total (N = 28)
Age (yr)	32.6 ± 6.59	31.2 ± 6.08	31.9 ± 6.27	30.1 ± 6.23	33.9 ± 6.20	32.1 ± 6.38
Male n (%)	9 (64.3)	9 (64.3)	18 (64.3)	8 (57.1)	7 (50.0)	15 (53.6)
Female n (%)	5 (35.7)	5 (35.7)	10 (35.7)	6 (42.9)	7 (50.0)	13 (46.4)
Height (cm)	165.13 ± 7.805	165.75 ± 10.732	165.44 ± 9.213	163.93 ± 8.455	164.77 ± 7.388	164.35 ± 7.803
Weight (kg)	63.29 ± 7.365	62.36 ± 9.462	62.83 ± 8.334	59.81 ± 8.518	62.96 ± 7.283	61.38 ± 7.940
BMI (kg/m^2^)	23.15 ± 1.347	22.59 ± 1.222	22.87 ± 1.294	22.19 ± 1.887	23.16 ± 1.862	22.68 ± 1.905

### Pharmacokinetic results

**FIGURE 2:**
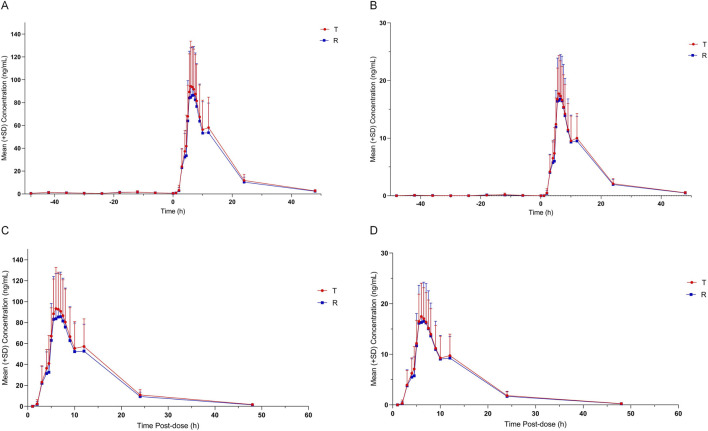
Arithmetic mean (±SD) plasma concentrations of VK_1_ E and Z isomer of T and R formulation under fasting condition versus time. **(A)** Measured value of VK_1_ E isomer. **(B)** Measured value of VK_1_ Z isomer. **(C)** Value of VK_1_ E isomer compared with baseline. **(D)** Value of VK_1_ Z isomer compared with baseline.

**FIGURE 3:**
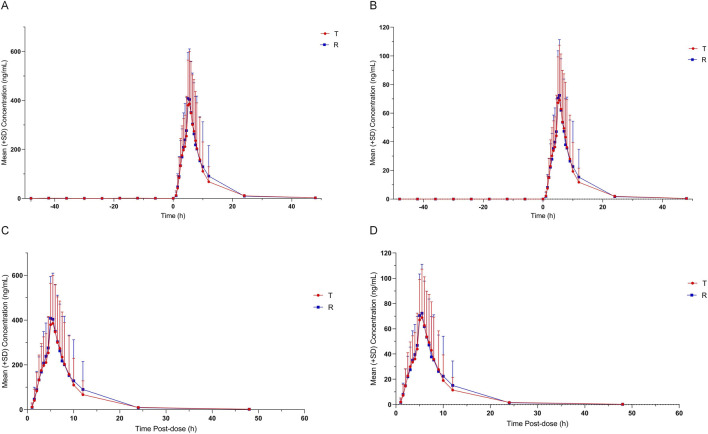
Arithmetic mean (±SD) plasma concentrations of VK_1_ E and Z isomer of T and R formulation under fed condition versus time. **(A)** Measured value of VK_1_ E isomer. **(B)** Measured value of VK_1_ Z isomer. **(C)** Value of VK_1_ E isomer compared with baseline. **(D)** Value of VK_1_ Z isomer compared with baseline.

The 9-day washout period was enough to meet the requirements of more than 5 times of t_1/2_. Before the second period of administration, the blood concentration of VK_1_ had returned to the level of baseline. In the fasting condition, healthy subjects received a single oral dose of 10 mg of the test formulation (Vitamin K_1_ Injection) and the reference formulation (KONAKION®MM), resulting in geometric mean ratios (GMRs) of the changes from baseline for C_max_, AUC_0-t_, and ${\text{AUC}}_{0-\infty }$ of the Vitamin K_1_ E isomer (test formulation/reference formulation) of 108.74%, 112.19%, and 113.92%, respectively, with corresponding 90% confidence intervals (CIs) of 99.17%–119.24%, 103.91%–121.13%, and 105.72%–122.77%, all of which fell entirely within the bioequivalence interval of 80.00%–125.00% (Table 2-5). In the fed condition, the GMRs for the changes from baseline of the E isomer’s C_max_, AUC_0-t_, and ${\text{AUC}}_{0-\infty }$ of 100.48%, 102.91%, and 102.18%, with 90% CIs of 83.56%–120.82%, 84.98%–124.63%, and 83.97%–124.32%, all of which also fell within the 80.00%–125.00% bioequivalence interval. These results collectively demonstrate that the test formulation was bioequivalent to the reference formulation under both fasting and fed conditions when administered orally. The presence of food significantly enhanced the systemic exposure of vitamin K_1_. Under fed conditions, the mean C_max_ and AUC_0-t_ values for the isomers were approximately 4–5 times higher and 2 to 3 times higher, respectively, compared to fasting conditions. There was also a slight decrease in median T_max_ (approximately 1.0–1.5 h earlier) under fed conditions, indicating a slightly faster absorption rate. For the Z isomer as a supporting data, the GMRs of the changes from baseline for C_max_, AUC_0-t_, and ${\text{AUC}}_{0-\infty }$ also all fell within the same bioequivalence interval under both fasting and fed conditions. Notably, the observed within-subject variability under fed conditions was higher than initially estimated, which is consistent with the known pharmacokinetic behavior of highly lipophilic vitamins, where food intake can introduce greater variability in absorption. The details were in Table 2–5; Figures 2, 3; Supplementary Figures S1, S2.

**TABLE 2:** Pharmacokinetic parameters of vitamin K_1_ E isomer compared with baseline.

Parameters	Fasting condition		Fed condition	
	Test formulation (Mean ± SD (CV%)), N = 28	Reference formulation (Mean ± SD (CV%)), N = 28	Test formulation (Mean ± SD (CV%)), N = 27^#^	Reference formulation (Mean ± SD (CV%)), N = 27^#^
C_max_ (ng/mL)	106.587 ± 36.279 (34.0)	100.884 ± 42.369 (42.0)	460.145 ± 212.599 (46.2)	473.671 ± 226.488 (47.8)
AUC_0-t_ (h·ng/mL)	1161.722 ± 446.009 (38.4)	1059.331 ± 457.544 (43.2)	2754.481 ± 1521.007 (55.2)	2862.842 ± 1974.826 (69.0)
${\text{AUC}}_{0-\infty }$ (h·ng/mL)	1180.746 ± 452.052 (38.3)	1068.689 ± 470.169 (44.0)	2775.367 ± 1525.173 (55.0)	2929.915 ± 1999.623 (68.2)
T_max_ (h)^*^	6.000 (5,9)	6.250 (3,12)	5.000 (3,6.5)	5.000 (4,12)
t_1/2z_ (h)	7.278 ± 0.901 (12.4)	7.114 ± 0.728 (10.2)	7.089 ± 1.421 (20.0)	6.645 ± 1.065 (16.0)
λ_z_ (1/h)	0.097 ± 0.015 (15.2)	0.098 ± 0.011 (10.7)	0.102 ± 0.023 (22.2)	0.107 ± 0.019 (17.9)
V_z_/F (L)	100.731 ± 36.311 (36.0)	114.505 ± 48.521 (42.4)	49.399 ± 28.972 (58.6)	46.167 ± 27.042 (58.6)
CL_z_/F (L/h)	9.515 ± 3.085 (32.4)	11.065 ± 4.397 (39.7)	4.677 ± 2.447 (52.3)	4.847 ± 3.130 (64.6)
AUC__%Extrap_ (%)	1.626 ± 0.645 (39.7)	1.478 ± 0.544 (36.8)	0.864 ± 0.444 (51.4)	0.805 ± 0.820 (101.9)

C_max_, maximum plasma concentration; AUC, area under the plasma concentration-time curve; ${\text{AUC}}_{0-\infty }$ , AUC from time 0 to infinity; AUC_0-t_, AUC from time 0 to t; T_max_, time to reach C_max_; t_1/2z,_ half-life time; λ_z,_ elimination rate constant; V_z_/F, apparent volume of distribution during the terminal phase; CL_z_/F, apparent clearance during the terminal phase; AUC__%Extrap_, percentage of the ${\text{AUC}}_{0-\infty }$ that is extrapolated beyond the last quantifiable concentration.

^*^ Data were presented as the median (range).

^#^ One subject withdrew from the study before receiving R drug, and one subject vomited within 2 times of the median T_max_ time after receiving T drug, which was not included in the pharmacokinetic parameter set.

**TABLE 3:** Pharmacokinetic parameters of vitamin K_1_ Z isomer compared with baseline.

Parameters	Fasting condition		Fed condition	
	Test formulation (Mean ± SD (CV%)), N = 28	Reference formulation (Mean ± SD (CV%)), N = 28	Test formulation (Mean ± SD (CV%)), N = 27^#^	Reference formulation (Mean ± SD (CV%)), N = 27^#^
C_max_ (ng/mL)	19.846 ± 5.856 (29.5)	19.229 ± 7.552 (39.3)	84.257 ± 40.138 (47.6)	84.988 ± 42.229 (49.7)
AUC_0-t_ (h·ng/mL)	199.711 ± 72.455 (36.3)	189.788 ± 82.395 (43.4)	477.716 ± 262.372 (54.9)	489.892 ± 318.184 (64.9)
${\text{AUC}}_{0-\infty }$ (h·ng/mL)^#^	202.534 ± 73.984 (36.5)	191.834 ± 85.735 (44.7)	480.659 ± 262.928 (54.7)	499.454 ± 322.892 (64.6)
T_max_ (h)^*^	6.000 (5,9)	6.000 (3,12)	5.500 (3,7.5)	5.500 (5,12)
t_1/2z_ (h)	6.697 ± 1.307 (19.5)	6.556 ± 1.096 (16.7)	6.184 ± 1.272 (20.6)	5.805 ± 0.985 (17.0)
λ_z_ (1/h)	0.108 ± 0.022 (20.4)	0.109 ± 0.023 (21.2)	0.117 ± 0.023 (19.9)	0.123 ± 0.023 (18.7)
V_z_/F (L)	519.392 ± 151.626 (29.2)	565.743 ± 207.991 (36.8)	235.354 ± 109.129 (46.4)	222.081 ± 113.350 (51.0)
CL_z_/F (L/h)	55.239 ± 17.805 (32.2)	61.974 ± 25.365 (40.9)	26.847 ± 13.844 (51.6)	27.486 ± 16.459 (59.9)
AUC__%Extrap_ (%)	1.275 ± 0.930 (72.9)	1.141 ± 0.653 (57.2)	0.715 ± 0.622 (87.0)	0.525 ± 0.615 (117.1)

C_max_, maximum plasma concentration; AUC, area under the plasma concentration-time curve; ${\text{AUC}}_{0-\infty }$ , AUC from time 0 to infinity; AUC_0-t_, AUC from time 0 to t; T_max_, time to reach C_max_; t_1/2z,_ half-life time; λ_z,_ elimination rate constant; V_z_/F, apparent volume of distribution during the terminal phase; CL_z_/F, apparent clearance during the terminal phase; AUC__%Extrap_, percentage of the ${\text{AUC}}_{0-\infty }$ that is extrapolated beyond the last quantifiable concentration.

^*^ Data were presented as the median (range).

^#^ One subject withdrew from the study before receiving R drug, and one subject vomited within 2 times of the median T_max_ time after receiving T drug, which was not included in the pharmacokinetic parameter set.

**TABLE 4:** Bioequivalence evaluation for the primary pharmacokinetic parameters of vitamin K_1_ E isomer.

Parameters	T/R ratio (%)	90% CI (%)	Inter-CV (%)
Fasting condition			
C_max_ (ng/mL)	108.74	99.17–119.24	32.23
AUC_0-t_ (h·ng/mL)	112.19	103.91–121.13	35.80
${\text{AUC}}_{0-\infty }$ (h·ng/mL)	113.92	105.72–122.77	36.19
Fed condition			
C_max_ (ng/mL)	100.48	83.56–120.82	46.25
AUC_0-t_ (h·ng/mL)	102.91	84.98–124.63	39.30
${\text{AUC}}_{0-\infty }$ (h·ng/mL)	102.18	83.97–124.32	38.51

C_max_, maximum plasma concentration; AUC, area under the plasma concentration-time curve; AUC_0-t_, AUC from time 0 to t; ${\text{AUC}}_{0-\infty }$ , AUC from time 0 to infinity.

**TABLE 5:** Bioequivalence evaluation for the primary pharmacokinetic parameters of vitamin K_1_ Z isomer.

Parameters	T/R ratio (%)	90% CI (%)	Inter-CV (%)
Fasting condition			
C_max_ (ng/mL)	106.73	97.12–117.30	29.69
AUC_0-t_ (h·ng/mL)	108.13	100.65–116.15	35.93
${\text{AUC}}_{0-\infty }$ (h·ng/mL)	109.13	101.44–117.41	36.39
Fed condition			
C_max_ (ng/mL)	102.43	85.98–122.03	47.64
AUC_0-t_ (h·ng/mL)	102.86	86.35–122.52	39.74
${\text{AUC}}_{0-\infty }$ (h·ng/mL)	102.46	85.61–122.63	39.23

C_max_, maximum plasma concentration; AUC, area under the plasma concentration-time curve; AUC_0-t_, AUC from time 0 to t; ${\text{AUC}}_{0-\infty }$ , AUC from time 0 to infinity.

### INR estimation

INR values remained within the normal range under both fasting and fed conditions, with only minor fluctuations observed within the normal range and no discernible upward or downward trend.

Under fasting condition, the measured AUEC_0–48h_ values for test and reference formulations were 53.473 ± 2.475 and 53.689 ± 2.082, while the changes from baseline for test and reference formulations were 0.572 ± 0.659 and 0.601 ± 0.682, respectively. Under fed condition, the measured AUEC_0–48h_ values for test and reference formulations were 52.221 ± 2.179 and 52.248 ± 1.696, with changes from baseline for test and reference formulations of 1.488 ± 1.140 and 1.397 ± 1.041, respectively. Overall, a single oral dose had a minimal impact on INR values, and the extent of INR changes was comparable between the reference and test formulations. The details were in Table 6.

**TABLE 6:** Pharmacodynamic parameters of INR compared with baseline after oral administration of test formulation and reference formulation under fasting and fed conditions.

Parameters	Fasting condition		Fed condition	
	Test formulation (Mean ± SD (CV%)), N = 28	Reference formulation (Mean ± SD (CV%)), N = 28	Test formulation (Mean ± SD (CV%)), N = 28	Reference formulation (Mean ± SD (CV%)), N = 27^#^
E_max_	0.035 ± 0.029 (83.9)	0.035 ± 0.030 (87.7)	0.057 ± 0.034 (59.7)	0.057 ± 0.035 (61.5)
AUEC_0–48h_	0.572 ± 0.659 (115.1)	0.601 ± 0.682 (113.4)	1.488 ± 1.140 (76.6)	1.397 ± 1.041 (74.5)
T_Emax_ (h)^*^	3.000 (0,48.02)	3.000 (0,48)	12.000 (0,48)	12.000 (0,48)

E_max_, maximum effect; AUEC_0–48h_, area under the effect-time curve from time 0–48h; T_Emax_, time to reach E_max_.

^*^ Data were presented as the median (range).

^#^ One subject withdrew from the study before receiving R drug, which was not included in the pharmacodynamic parameter set.

### Safety

Under the fasting condition, six subjects (21.4%) experienced 12 AEs, all of which were mild (Grade 1) and resolved or recovered. After taking the test formulation, three subjects (10.7%) experienced 7 AEs, with three classified as drug-related adverse events (ADRs), while four subjects (14.3%) experienced 5 AEs, with four classified as ADRs after taking the reference formulation. No AEs led to withdrawal or were considered serious adverse events (SAEs). Under the fed condition, nine subjects (32.1%) experienced 11 AEs. After taking the test formulation, five subjects (17.9%) experienced 7 AEs, including six classified as ADRs, with one AE leading to withdrawal and being Grade 2 in severity, and six being Grade 1. Six AEs resolved or recovered, and one outcome was unknown. After taking the reference formulation, four subjects (14.8%) experienced 4 AEs, all classified as ADRs and Grade 1, with three resolving or recovering and one outcome unknown. No SAEs occurred. The details were in Supplementary Table S1.

## Discussion

Vitamin K_1_ is widely present in foods, and its deficiency can lead to hemorrhagic disorders. Various routes of administration are commonly used in clinical practice. For the prevention of VK_1_ deficiency-induced bleeding in newborns, oral or intramuscular administration can be employed, while intravenous administration is often used in emergency situations in the ICU. Therefore, according to the requirements of China’s Center for Drug Evaluation (CDE), bioequivalence studies must be conducted for all three administration routes. This study focused on the oral administration route. To our knowledge, this was the first study to compare the oral bioequivalence of a Chinese-produced VK_1_ injection and the original drug KONAKION®MM under both fasting and fed conditions. Under fasting conditions, the 90% CIs for the GMRs of C_max_, AUC_0-t_, and ${\text{AUC}}_{0-\infty }$ for the VK_1_ E isomer were 99.17%–119.24%, 103.91%–121.13%, and 105.72%–122.77%, respectively. Under fed conditions, the corresponding 90% CIs were 83.56%–120.82%, 84.98%–124.63%, and 83.97%–124.32%. The results showed that the primary pharmacokinetic parameters of the VK_1_ E isomer from both formulations met the 80.00%–125.00% equivalence criteria, establishing their bioequivalence.

VK_1_ is an endogenous compound. To account for the background levels of VK_1_, we measured baseline VK_1_ levels over 48 h and subtracted the average baseline VK_1_ concentration from post-dose measurements. The study results indicated low and stable baseline VK_1_ plasma concentrations. After deducting the baseline concentration, post-dose VK_1_ plasma concentration profiles exhibited typical pharmacokinetic characteristics observed after oral administration of conventional drugs, with a T_max_ of 6 h and an elimination half-life of 7 h. A previous study (van Rein et al., 2014) reported a T_max_ of approximately 6 h in healthy subjects following oral administration of a vitamin K_1_ solution, a finding that was generally consistent with the results of our study. Additionally, the pharmacokinetic parameters observed following administration of the reference formulation in this study were generally consistent with those documented in prior studies of KONAKION®MM. Compared with the previous study with a 2 mg dosage, the C_max_ and AUC exhibited an approximately proportional relationship (Marinova et al., 2013).

In this study, the C_max_ and AUC after oral administration under fed conditions were significantly higher than those under fasting conditions. The solubilizing agents in the VK_1_ formulation are glycocholic acid and phospholipids, both of which are also components of bile. When administered under fasting conditions, these excipients play a certain role in the dissolution and absorption of the drug. However, when administered in the fed state, bile secretion increases, and the formation of mixed bile salt micelles significantly enhances the dissolution and absorption of VK_1_. Additionally, the high fat content in the meal further promotes the solubilization of VK_1_. Moreover, the delayed gastric emptying caused by the high-fat meal allows for a slower entry of VK_1_ into the intestinum tenue, avoiding saturable absorption mechanisms and thereby enabling more complete drug absorption. A previous study (Gijsbers et al., 1996) has shown that fat enhances the bioavailability of vitamin K_1_, which is consistent with the findings of this study. We recommend that the effect of food be stated in the product label.

VK_1_ is an organic compound that exhibits stereoisomerism, a phenomenon also observed in other drugs, such as trans-ethinylestradiol, which has pharmacodynamic effects, whereas its cis form has minimal activity. For VK_1_, the E isomer (trans configuration) is the primary pharmacologically active component, and thus the main endpoints of this study were the C_max_, AUC_0-t_, and ${\text{AUC}}_{0-\infty }$ of the VK_1_ E isomer. The analytical methods used in this study separately quantified the E and Z isomers. The data of Z isomers was considered as supplementary data. The result showed that the equivalence evaluation of both isomers met the standard of 80.00%–125.00%, which reflected the consistency of the two formulations.

This study has some limitations. Firstly, one of the indications for VK_1_ is the prevention of VK deficiency-induced bleeding in newborns; however, due to ethical considerations regarding vulnerable populations, only healthy adult subjects were included in this study. Therefore, the PK characteristics of VK_1_ in newborns have not been established. Furthermore, future studies conducted in the general population or in patients with hepatic or renal impairment will help to further understand the pharmacokinetic characteristics of VK_1_. Secondly, although this study explored changes in INR, no significant changes were observed in healthy individuals, and further studies are needed to evaluate the pharmacodynamic effects in patients with VK_1_ deficiency and coagulation abnormalities. However, this study confirmed that in individuals with normal coagulation function, therapeutic doses of orally administered VK_1_ do not significantly alter INR levels. In the future, additional indicators may be explored using novel technologies, such as employing electrochemical probes to detect mitochondrial oxidative stress for assessing the effects of VK_1_ on target organs, or utilizing advanced delivery systems to achieve targeted oral delivery of VK_1_ (Yao et al., 2026; Hao et al., 2026). Thirdly, in view of the relatively small sample size, this study did not explore gender differences or age differences.

## Conclusion

Under both fasting and fed conditions, the bioequivalence was established after oral administration of 10 mg VK_1_ injection and the same dose of original drug KONAKION®MM. Furthermore, oral administration of VK_1_ is safe and tolerable.

## Data Availability

The original contributions presented in the study are included in the article/Supplementary Material, further inquiries can be directed to the corresponding author.

## Supplementary material

SUPPLEMENTARY FIGURE S1 Semi-logarithm curve of mean (±SD) plasma concentrations of VK_1_ E and Z isomer of T and R formulation under fasting condition versus time. **(A)** Measured value of VK_1_ E isomer. **(B)** Measured value of VK_1_ Z isomer. **(C)** Value of VK_1_ E isomer compared with baseline. **(D)** Value of VK_1_ Z isomer compared with baseline.

SUPPLEMENTARY FIGURE S2 Semi-logarithm curve of mean (±SD) plasma concentrations of VK_1_ E and Z isomer of T and R formulation under fed condition versus time. **(A)** Measured value of VK_1_ E isomer. **(B)** Measured value of VK_1_ Z isomer. **(C)** Value of VK_1_ E isomer compared with baseline. **(D)** Value of VK_1_ Z isomer compared with baseline.
