# Supplementary figures and images for: A randomized, open, two-period, cross-over, single oral dose, pharmacokinetics and bioequivalence study of vitamin K1 in healthy volunteers under fasting and fed conditions
Source: Front Pharmacol. 2026 May 13;17:1802709. doi: 10.3389/fphar.2026.1802709 (PMC13213390; doi:10.3389/fphar.2026.1802709)

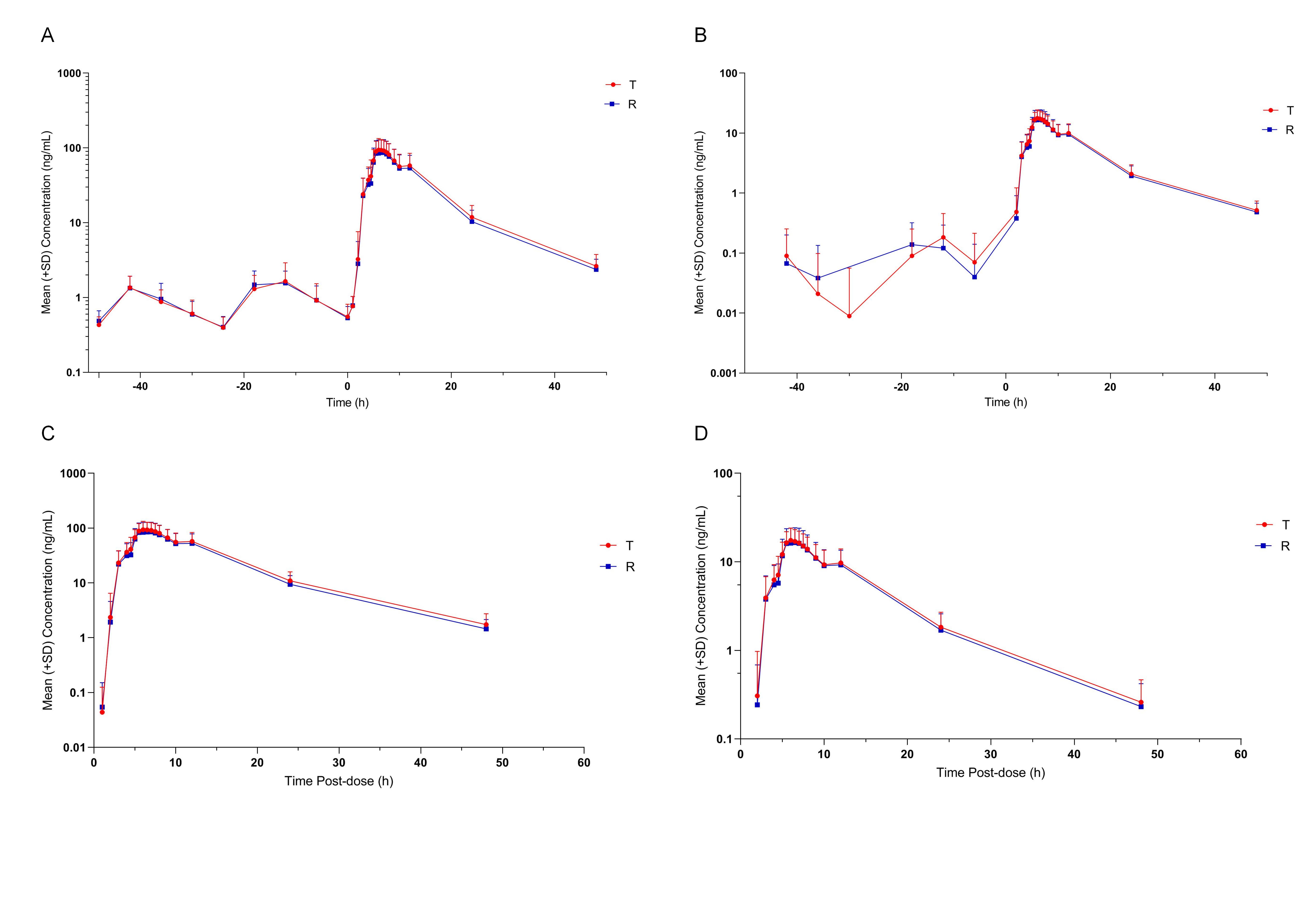

Supplement: Supplementary file 1 [file Image1.jpeg]

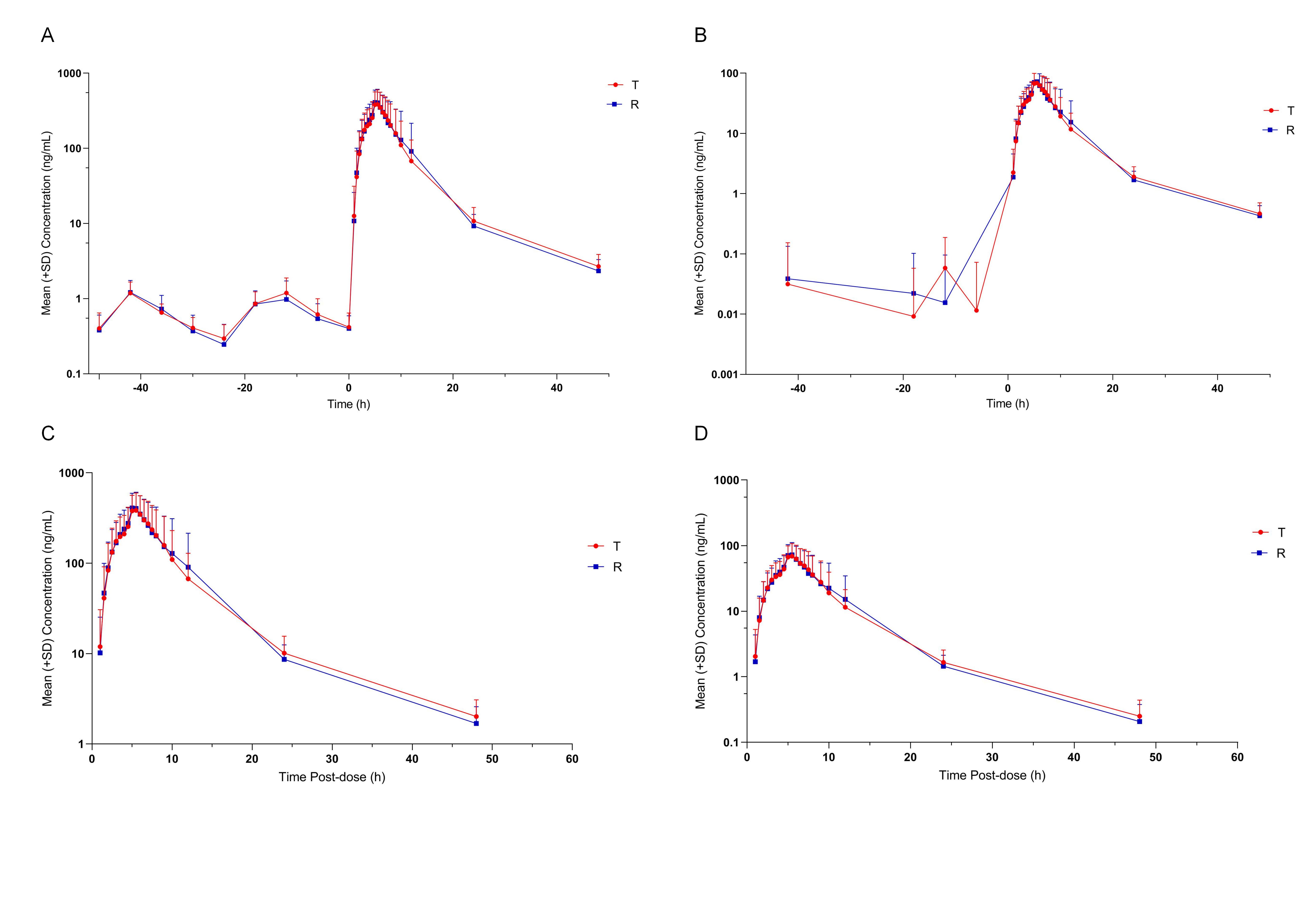

Supplement: Supplementary file 2 [file Image2.jpeg]
